# Gene Set-Based Functionome Analysis of Pathogenesis in Epithelial Ovarian Serous Carcinoma and the Molecular Features in Different FIGO Stages

**DOI:** 10.3390/ijms17060886

**Published:** 2016-06-06

**Authors:** Chia-Ming Chang, Chi-Mu Chuang, Mong-Lien Wang, Ming-Jie Yang, Cheng-Chang Chang, Ming-Shyen Yen, Shih-Hwa Chiou

**Affiliations:** 1 Institute of Oral Biology, National Yang-Ming University, Taipei 112, Taiwan; cm_chang@vghtpe.gov.tw; 2 School of Medicine, National Yang-Ming University, Taipei 112, Taiwan; cmjuang@gmail.com (C.-M.C.); monglien@gmail.com (M.-L.W.); mjyang@vghtpe.gov.tw (M.-J.Y.); msyen@vghtpe.gov.tw (M.-S.Y.); 3 Department of Obstetrics and Gynecology, Taipei Veterans General Hospital, Taipei 112, Taiwan; 4 Institute of Clinical Medicine, School of Medicine, National Yang-Ming University, Taipei 112, Taiwan; 5 Department of Medical Research, Taipei Veterans General Hospital, Taipei 112, Taiwan; 6 Department of Obstetrics and Gynecology, Tri-Service General Hospital, National Defense Medical Center, Taipei 112, Taiwan; sundoor66@gmail.com; 7 Department & Institute of Pharmacology, National Yang-Ming University, Taipei 112, Taiwan

**Keywords:** epithelial serous ovarian cancer, function, integrative analysis, gene expression microarray, gene set, machine learning

## Abstract

Serous carcinoma (SC) is the most common subtype of epithelial ovarian carcinoma and is divided into four stages by the Federation of Gynecologists and Obstetrics (FIGO) staging system. Currently, the molecular functions and biological processes of SC at different FIGO stages have not been quantified. Here, we conducted a whole-genome integrative analysis to investigate the functions of SC at different stages. The function, as defined by the GO term or canonical pathway gene set, was quantified by measuring the changes in the gene expressional order between cancerous and normal control states. The quantified function, *i.e.*, the gene set regularity (GSR) index, was utilized to investigate the pathogenesis and functional regulation of SC at different FIGO stages. We showed that the informativeness of the GSR indices was sufficient for accurate pattern recognition and classification for machine learning. The function regularity presented by the GSR indices showed stepwise deterioration during SC progression from FIGO stage I to stage IV. The pathogenesis of SC was centered on cell cycle deregulation and accompanied with multiple functional aberrations as well as their interactions.

## 1. Introduction

Epithelial ovarian cancers (EOC) are classified into several subtypes of heterogeneous diseases. Serous carcinoma (SC) is the most common subtype of EOCs, accounting for approximately 70% of them [1], and has a poor prognosis with a five-year survival rate of only 10%–20%. Based on findings through surgical staging, the Federation of Gynecologists and Obstetrics (FIGO) system [2], the most commonly utilized staging system, divides SC into four stages: stage I: tumor confined to ovaries; stage II: tumor involves one or both ovaries with pelvic extension; stage III: tumor involves one or both ovaries with cytologically or histologically confirmed spread to the peritoneum outside the pelvis and/or metastasis to the retroperitoneal lymph nodes; and stage IV: distant metastasis. The prevalence of stages I, II, III, and IV was 10.3%, 8.4%, 55% and 26.3% of total SC cases, respectively [3]. FIGO staging was established based on disease progression, including the primary site, lymph nodal draining and metastatic sites. A considerable number of clinical studies have shown its applicability to evaluate disease survival or the treatment response for SC. 

As a complex disease, the carcinogenesis of SC evolves in a number of aberrant functions, and these functions fluctuate with disease progression. Knowing how these functions deteriorate from SC stage I to IV will facilitate the investigation of SC pathogenesis. Although the FIGO staging system shows great consistence with the progression and disease severity of SC, it does not provide the information about the regularity of cellular functions at different stages. Currently, the relationship between the molecular functions or biological processes with different FIGO stages of SC has not been measured. In this study, we conducted a gene set-based study to investigate and quantify the molecular features of SC at different FIGO stages. This study integrated microarray gene expression datasets from a publicly available database by converting them to gene expression orderings through the gene ontology (GO) term or canonical pathway gene sets from the Molecular Signatures Database (MSigDB) [4]. The GO term gene set database collected 1454 gene sets defining biological processes, molecular functions or cellular components; the canonical pathway gene set database collected 1330 canonical pathways from the Kyoto Encyclopedia of Genes and Genomes (KEGG), Pathway Interaction Database (PID), Reactome databases, *etc.* For simplicity, we refer to the molecular function, biological process, cellular component and pathway defined by a gene set as a “function” in this study. Currently, no databases can annotate the functionome, *i.e.*, all the biological functions in human bodies. We utilized the two databases to annotate human functionome because they collected a relatively comprehensive set of human functions. These functions were quantified by measuring the change in the gene expression ranking between cancerous and normal states in a given gene set. This quantified change in the gene expression ranking in a gene set was defined as the “gene set regularity (GSR) index”, which measures the regularity of the function defined by that gene set. Then, the pathogenesis of SC at different stages was evaluated with the GSR indices using statistical methods, set analysis and exploratory factor analysis (EFA) to identify the most important deregulated functions and the interaction network contributing to SC carcinogenesis. 

## 2. Results

### 2.1. DNA Microarray Gene Expression Datasets and Gene Sets

A total of 1236 samples were initially collected from the Gene Expression Omnibus (GEO) database, and 1029 samples remained in this study after the datasets that did not meet the criteria were removed. The final dataset included 34, 39, 689, and 131 samples for SC stages I to IV, respectively, as well as 136 normal control samples, as shown in Table 1. These data were collected from 35 datasets containing five different DNA microarray platforms without missing data. Detailed information about the samples, including the staging, DNA microarray platform, dataset series and accession numbers, are presented in Table S1. The definitions of the gene sets were downloaded from the MSigDB (versions: “c5.all.v5.1.symbols.gmt” and “c2.cp.v5.1.symbols.gmt”) for the GO term and canonical pathway gene sets, which contained 1454 and 1330 gene set definitions. Because different genes were utilized in different platforms, 1443, 1442, 1377 and 1440 GO gene sets and 1324, 1323, 1269 and 1322 canonical pathway gene sets were ultimately utilized for the stage I–IV groups.

### 2.2. Means and Histograms of the Gene Set Regularity (GSR) Indices for the Four Stage Groups

**Figure 1:**
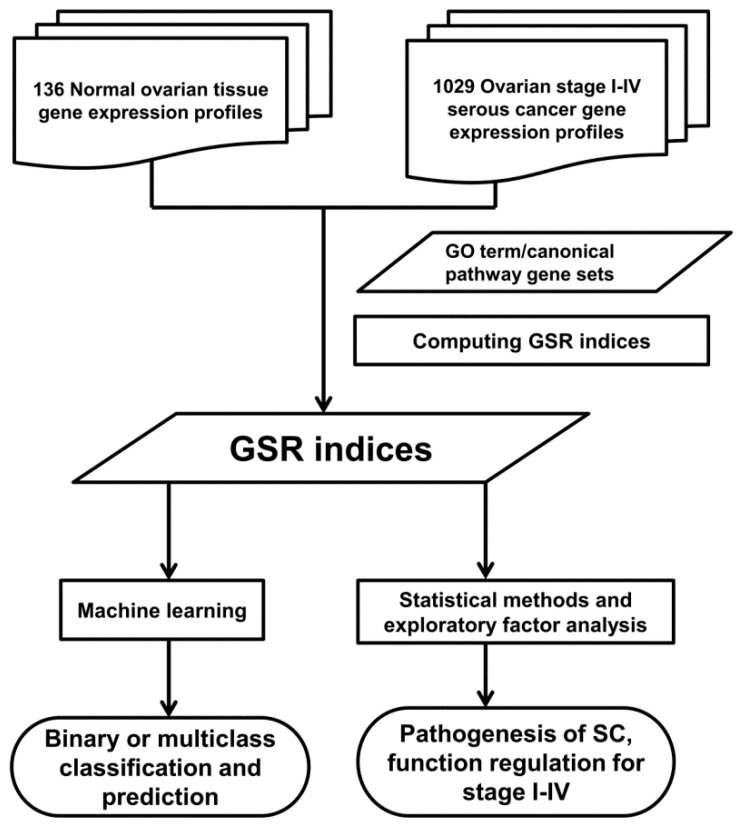
Workflow of the gene set regularity model. The gene set regularity (GSR) index was computed by converting the gene expression ordering of each sample in each group using the gene ontology (GO) term or canonical pathway gene set. A machine-learning algorithm was trained to recognize the patterns consisting of the GSR index matrices and then executed the binary (each stage *vs.* control group) or multiclass (stage I to IV + control groups) classifications. The functionome analyses were performed to investigate the pathogenesis of ovarian serous carcinoma (SC) using statistical methods, hierarchical clustering and exploratory factor analysis.

The workflow of the GSR model is displayed in Figure 1 and described in detail in the Materials and Methods section. The GSR index ranged from 0 to 1, where 1 represented no changes in the gene expression ordering between the SC and the most common gene expression orderings in the normal controls, and 0 represented completely different gene expression orderings from the normal state, meaning the most chaotic state of gene set regularity. The informativeness of the GSR index was evaluated by the accuracies of classification and predication using machine learning and the functionome patterns generated from the 1454 GO terms or 1330 canonical pathway gene sets.

The differences in the GSR indices between each stage and the normal control group were statistically significant (*p* < 0.05, Table 1), indicating that the functions were generally deregulated in the SC group compared with the normal control group. As shown in Table 1, the averages of the GSR indices decreased linearly from 0.7425 in stage I, to 0.7088 in stage II, 0.6483 in stage III and ultimately 0.6197 in stage IV, and the differences between two consecutive stage groups were also statistically significant, indicating that the functional regulation deteriorated steadily from stage I to IV. 

**Figure 2:**
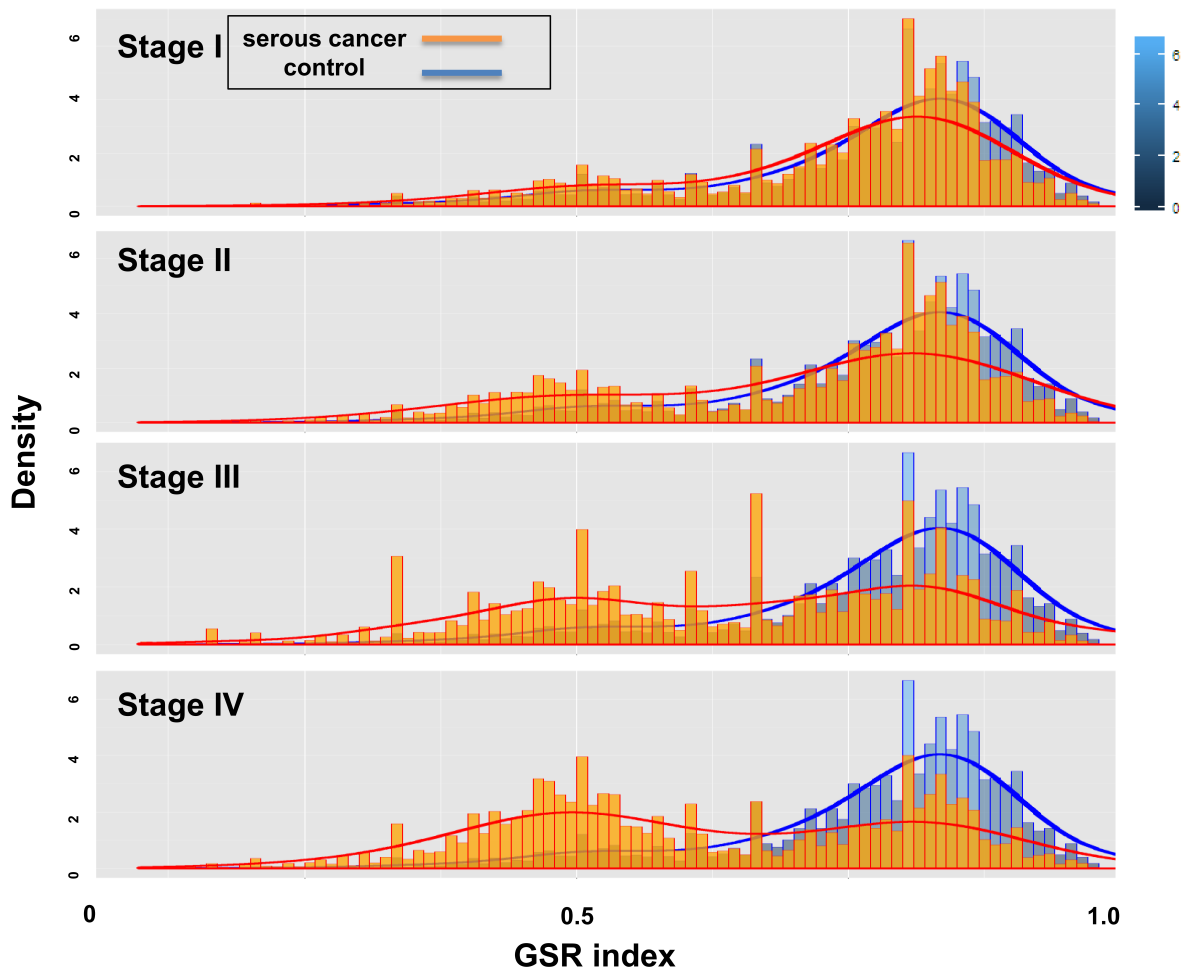
Histograms of the gene set regularity indices for the stage I–IV and control groups. The figures show the distributions of the GSR indices from the SC stage I–IV and control groups. The normal control group (**blue**), which is located on the right side of the histogram, was the same for the four stage groups. A second group of smaller GSR indices, which is located on the left side, was observed and increased in density from stage I to IV (**orange**).

When displayed on the histogram (Figure 2), the GSR indices of each stage and control group appeared to be overlapping, but they have different distributions. Compared with the same control group, the distribution of the stage I group was similar to the control group, whereas a second group of smaller GSR indices, which are located on the left side, appeared and grew in density from stage II to IV. This result indicated that a group of deregulated functions existed and increased in number during disease progression. 

### 2.3. The Relationship of the Four Serous Carcinoma (SC) Stage Groups Revealed by Hierarchical Clustering

**Figure 3:**
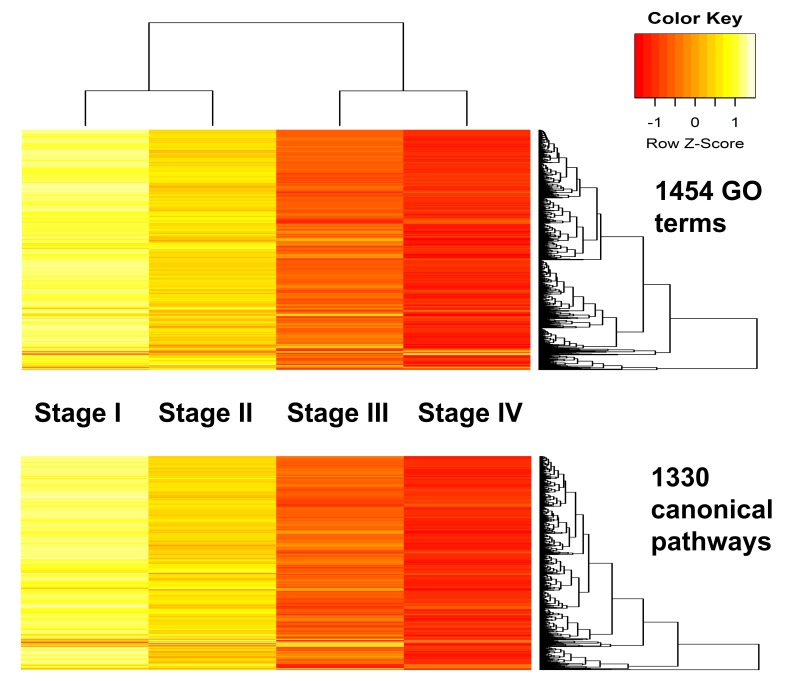
Heatmaps and dendrogram for the stage I–IV groups. The dendrogram (top of the heatmap) show the relationship between the four stage groups. When displayed on the heatmaps, the GSR indices of the four stages computed through either the GO terms or canonical pathway gene sets showed distinct patterns and stepwise deteriorations in the functions from stage I to IV.

Unsupervised classification by hierarchical clustering was utilized to uncover the relationship between the four stages and the unlabeled GSR indices. Based on function regularity, the order of stages I to IV could be accurately recognized in the dendrogram (Figure 3). When displayed on the heatmaps, the GSR indices of the four stages showed stepwise deteriorations in the functions that were compatible with the severity of SC from stage I to IV. These findings indicated the GSR indices could provide sufficient information to make a clear distinction among the four stage groups. It also provided the evidence that the progression of SC stages I to IV classified by the FIGO staging system was compatible with the severity of function regularity, as quantified by the GSR model.

### 2.4. Function Regularity Patterns among the Four Stages Classified and Predicted by Machine Learning

Because distinct function regularity patterns were observed among the four stages of SC, as shown in the histograms, we utilized machine learning to recognize, classify and predict the patterns to evaluate the informativeness of the GSR indices. Supervised classification was performed by support vector machine (SVM), and the performance was assessed by determining the accuracies of the binary and multiclass classifications. The performance was tested by five-fold cross-validation. The results showed the highest accuracy of 99.43% in stage IV and the lowest accuracy of 98.82% in the stage I group. The areas under the curves (AUCs) ranged from 0.9692 to 0.9942 (Table 2). The accuracy of the multiclass classification among the stage I–IV groups was 90.38%. This decreased accuracy probably arose from the similarities in the functional regularity among the stage I–IV groups. These results revealed that the functions, as quantified by the GSR indices converted from the microarray gene expression profiles, can provide sufficient information for machine learning to recognize and perform adequate recognition and classification. These results also indicated the GSR indices could be utilized for molecular classification among gene expression profiles from different FIGO stages of SC. 

### 2.5. The Most Significantly Deregulated Gene Ontology (GO) Terms and Canonical Pathways

The 1454 GO terms or 1330 canonical pathways among the four stages of SC groups were ranked by their *p* values to show the most deregulated functions at different stages of SC. Table 3 displays the 15 most deregulated GO terms for the stage I–IV groups; all the *p* values were significant. The top deregulated GO term for each stage group was “calcium channel activity”, “lysosomal membrane”, “protein tyrosine kinase activity” and “lysosomal membrane”. Lysosomal membrane was also the fifth most deregulated GO term for the stage I group. The other important deregulated GO terms for the stage I and II groups were those functions related to channel activity, transport, binding, metabolism, cell development and maturation. Noticeably, the proportion of cell cycle-related GO terms increased dramatically in stages III and IV. The 15 most deregulated canonical pathways for stages I to IV are displayed in Table 4; all of the *p* values were significant. The top deregulated pathway for each stage group was “Reactome CD28-dependent phosphoinositide 3-kinase-AKT (PI3K-AKT) signaling”, “Biocarta A Kinase Anchor Protein 13 (AKAP13) pathway”, “PID androgen receptor transcription factor (AR TF) pathway” and “KEGG glycosphingolipid biosynthesis ganglio series”. The full list of GO terms and canonical pathways, as well as the corresponding *p* values, are shown in Tables S2 and S3.

### 2.6. The Commonly Deregulated GO Terms and Canonical Pathway Gene Sets among the Four Stages

**Figure 4:**
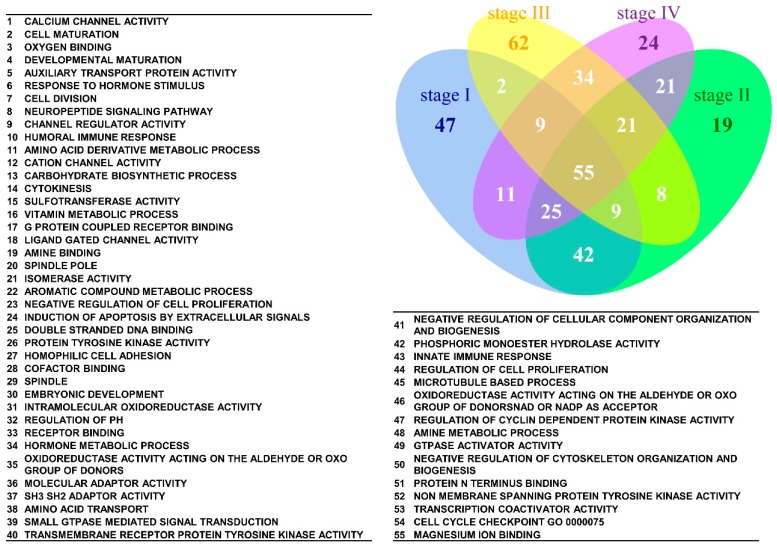
Venn diagram of the 200 most significantly deregulated GO terms for the stage I–IV groups. The results of the set analysis of the stage I–IV groups showing the 200 most significantly deregulated GO terms ranked by their *p* values are displayed on the Venn diagram to show the gene set numbers of all possible logical relations among the stage I–IV groups. The 55 most commonly deregulated GO terms among the four groups are listed.

**Figure 5:**
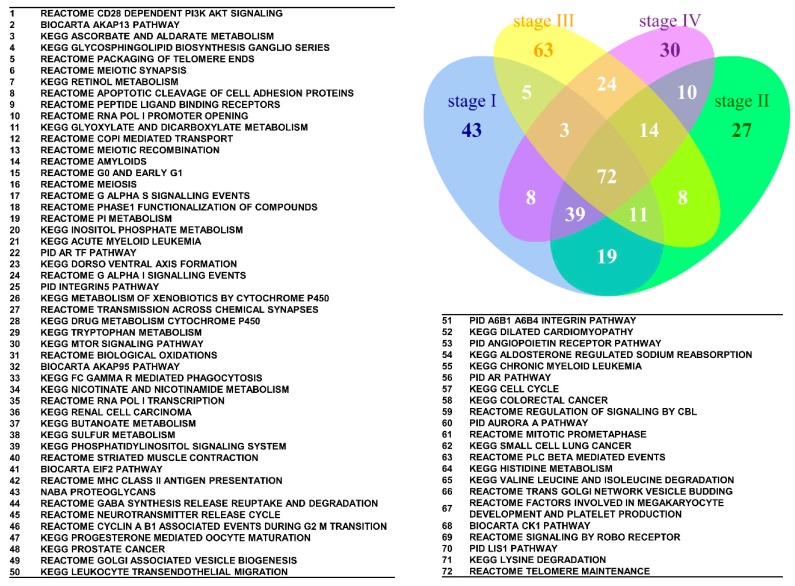
Venn diagram of the 200 most significantly deregulated canonical pathways for the stage I–IV groups. The results of the set analysis of the stage I–IV groups showing the 200 most significantly deregulated canonical pathways ranked by their *p* values are displayed on the Venn diagram to show the gene set numbers of all possible logical relations among the stage I–IV groups. The 72 most commonly deregulated canonical pathways among the four groups are listed.

As shown in Table 3, certain GO terms clearly co-occurred among the four stages, indicating the interaction of deregulated functions in the pathogenesis of SC. To discover the members of the interaction network, we utilized set analysis to identify the commonly deregulated gene sets among the stage I–IV groups. The 200 most deregulated GO term or canonical pathway gene sets for each group were selected for set analysis; all the *p* values were significant. There were 55 commonly deregulated GO terms among the stage I–IV groups, as shown in Figure 4. Based on the GO hierarchy, the 55 GO terms could be summarized in the following categories: cell cycle (“cell division”, “cytokinesis”, “spindle”, “double-stranded DNA binding”, and “cell cycle check point”), channel activity (“calcium channel activity” and “ligand-gated channel activity”), hormone response, metabolism, protein kinase activity, oxidoreductase activity, GTPase activity and binding (“oxygen binding”, “receptor binding” and “amine binding”). Figure 5 shows the results of the set analysis and the commonly deregulated canonical pathways. There were 72 commonly deregulated canonical pathways among the four stages. The results revealed that a relatively large proportion of these deregulated pathways were related to cell cycle, such as “Reactome meiotic synapsis”, “Reactome RNA Pol I promoter opening”, “Reactome G0 and early G1”, “Biocarta eukaryotic initiation factor-2 (EIF2) pathway”, “KEGG cell cycle”, “Reactome mitotic prometaphase”, and “Reactome telomere maintenance”. The other important commonly deregulated pathways included the PI3K-AKT, AKAP13, metabolism, NOTCH and mammalian target of rapamycin (mTOR) signaling pathways.

### 2.7. The Elements of Serous Carcinoma Carcinogenesis Networks Discovered by Exploratory Factor Analysis (EFA)

EFA can detect the underlying structure among numerous gene set variables; therefore, we performed EFA to discover the elements involved in the networks of SC carcinogenesis among these deregulated GO terms. For simplicity, we merged all of the datasets together, recomputed the GSR indices, and then executed the EFA. The EFA revealed eight factors, indicating eight groups of elements involved in the pathogenesis networks. In brief, factor 2 contained the elements related to channel activity and protein tyrosine kinase activity; factor 3 was related to actin and the cytoskeleton; factor 4 was related to protein complex assembly and cell maturation; factor 5 was related to oxidoreductase activity, cell adhesion and DNA binding; factor 6 was related to the cell cycle; factor 7 was related to cell adhesion and binding; factor 8 belonged to one part of factor 2; and factor 1 combined factor 2 and 8, as well as the following GO terms: metabolism, catabolism, cell development/differentiation, programmed cell death, cell proliferation, immune response, and regulation of transcription. These deregulated functions contributed to carcinogenesis and participated in the interaction networks of SC. Factor 1 was the main network, and the other factors were its sub-networks. The full list of these factors and elements was presented in Table S4. 

### 2.8. Trees of Deregulated Gene Ontology Terms for Serous Carcinoma

**Figure 6:**
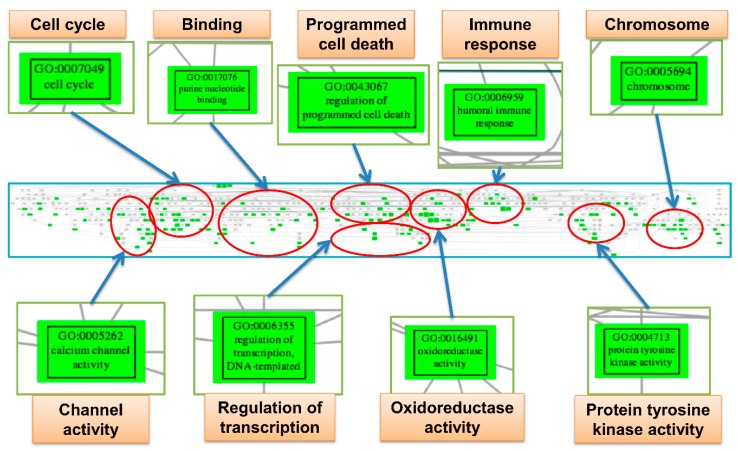
The gene ontology tree of serous carcinoma. This figure displayed a screenshot of the full gene ontology (GO) tree for serous carcinoma (SC) (**middle**). After mapping to the GO tree, the similar or related GO terms were clustered together. Each cluster was circled (**red**), and the important deregulated GO terms (**green boxes**) in the cluster were magnified to view the details. Each cluster was labeled by the common parental GO term (**orange rectangle**).

There were total 310 gene set elements among the eight factors revealed by EFA. To further summarize these elements, we remapped the 310 GO terms to establish the GO tree based on the parent-child relationship of GO hierarchies (Figure 6). When displayed on the GO tree, the redundant or related GO terms were summarized and visualized in an intuitive way. The related GO terms clustered together; each cluster was summarized according to their common parental GO term, including cell cycle, binding, programmed cell death, immune response, chromosome, channel activity, regulation of transcription, oxidoreductase activity and protein tyrosine kinase activity. The GO tree was consistent with the results of the EFA and further provided a more concise way to summarize the numerous deregulated GO terms. The full GO tree is presented in Figure S1. 

### 2.9. Interaction Network of SC Pathogenesis 

**Figure 7:**
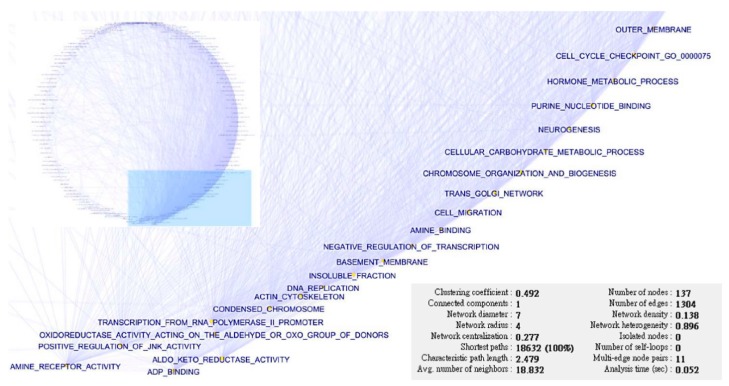
Interaction network of SC pathogenesis. The figure shows the interactions among the deregulated GO functions constructed from the elements identified in the exploratory factor analysis (EFA). The largest network consisting of 137 elements was extracted and displayed by the degree sorted circular layout. The deregulated functions with largest numbers were magnified to show the details. The network statistics are displayed in the bottom right part of the figure.

To show the interaction among the 310 gene set elements among the eight factors analyzed by the EFA, the interaction network was reconstructed based on the mutual information. We extracted and displayed the largest network consisting of 137 GO terms using Cytoscape (version 3.3.0) with the “degree sorted circular layout” (Figure 7). As a complex disease, the deregulated functions of SC exhibited extensive interactions; they affected each other and participated in the pathogenesis network of SC.

### 2.10. The Progressively Deregulated Functions in the Pathogenesis of SC from Stage I to IV

**Figure 8:**
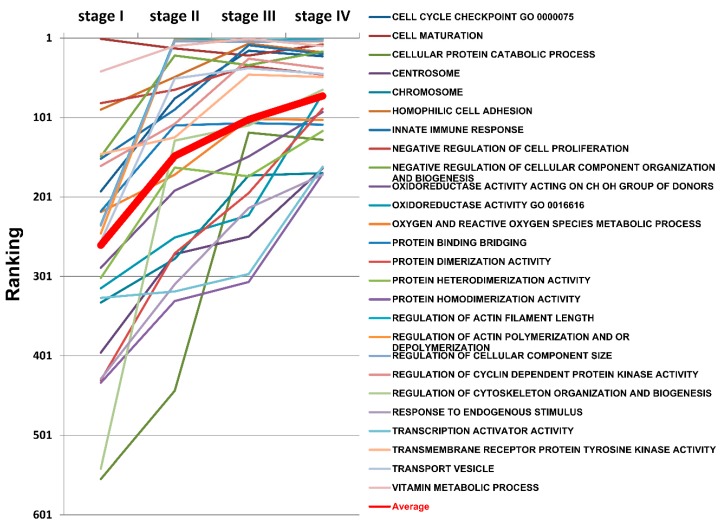
The rankings of the progressively deregulated GO terms from SC stage I to IV. The GO terms that were statistically significant and moved upward in rankings from SC stage I to IV were selected; a total of 26 GO terms met the criteria. The paths of the changes in ranking from stage I to IV of these progressively deregulated GO terms is displayed on the line chart.

The importance of given functions can be evaluated and compared by tracing their positions in the functionome during disease progression from stage I to IV. To filter the important deregulated functions in the pathogenesis of SC, those statistically significant GO terms that moved up in rankings from stage I to IV were selected, and the paths of ranking are displayed on the line chart shown in Figure 8. There were 26 GO terms that met the selection criteria; these GO terms were progressively deregulated and played increasingly important roles in the pathogenesis of SC from stage I to IV. These GO terms could be summarized in the following categories: cell cycle, cell proliferation and maturation, cell adhesion, immune response, oxidoreductase activity, binding, protein complex assembly, regulation of cytoskeleton organization, transport and metabolism. 

### 2.11. Differentially Expressed Genes in Ovarian Serous Carcinoma

To discover the differentially expressed genes (DEGs) in SC, we merged all microarray gene expression datasets and carried out integrative analysis for microarray gene expression datasets. The number of common genes among all of the datasets was 4686; these gene expressions for each of 1026 cases (892 SC and 134 control samples) were rescaled to cumulative proportion before integration. Table 5 and Table 6 listed the top 10 down-regulated and up-regulated genes, the related GO terms or canonical pathways, and the adjusted *p* values. The GO terms and pathways related to the DEGs were extracted from the GeneCards database (http://www.genecards.org/). The top 10 down-regulated genes were related to metabolism, catabolism, translation, apoptosis, cell proliferation, oxygenase activity, Notch signaling pathway, protein binding and metalloendopeptidase inhibitor activity. The top 10 up-regulated genes were related to transcription, p53 binding, cell cycle, apoptosis, mRNA processing, transport, metabolism, MAPK, ERBB2 and TGF-beta receptor signaling pathway. The full table list of these DEGs and their *p* values is presented in Table S5. To discover the progressively deregulated DEGs in the pathogenesis of SC from stage I to IV, we carried out integrative analysis for the microarray gene expression datasets for stage I to IV separately. The number of common genes among the datasets for the four stages was 4548. Those statistically significant DEGs that moved up in rankings from stage I to IV were selected and ordered by their ranking difference between stage IV and I. A total of 182 DEGs met the selection criteria and the top 20, as well as those related to GO terms or pathways, are listed in Table 7. These DEGs were progressively deregulated and played increasingly important roles in the pathogenesis of SC from stage I to IV. These DEGs could be summarized in the following categories: transcription, DNA binding, G-protein activity, GTPase activity and metabolism. The full table list of the progressively deregulated DEGs is presented in Table S6. These findings were consisted with, and provided an explanation for, the results computed through the GSR model.

## 3. Discussion

After converting the data to the GSR indices, the gene expression profiles of SC from stage I to IV showed clear stepwise patterns of deteriorating functions. The averages of the GSR indices revealed a linear decrease in their levels from stage I to IV. The histogram of each stage group showed two distributions of the GSR indices during disease progression. In addition to the normal functions, a second group of deregulated functions was observed beginning at stage I, and the indices for the members in this group increased as the disease progressed. These findings indicated the presence of a group of deregulated functions that increased in severity and number from stage I to IV. The subsequent analyses in the study were executed to investigate these deregulated functions and the pathogenesis of SC. The patterns of function regulation from stage I to IV could be accurately recognized and classified by unsupervised classification with hierarchical clustering and by supervised classification using SVM. The results showed that the informativeness of the GSR indices was sufficient to make a clear distinction among the four FIGO stages. 

The most deregulated GO terms in SC ordered by statistical significance were “calcium channel activity”, “lysosomal membrane”, “protein tyrosine kinase activity” and “lysosomal membrane”; the most deregulated canonical pathways were “Reactome CD28-dependent PI3K-AKT signaling”, “Biocata AKAP13 pathway”, “PID AR TF pathway” and “KEGG glycosphingolipid biosynthesis gangio series” for the stage I to IV groups, respectively. Channel activity is involved in the cell cycle control in the carcinogenesis of EOC [5]. The lysosome is an organelle responsible for autophagy and apoptosis, and the permeability of the lysosomal membrane is involved in the processes of carcinogenesis [6]. “Receptor tyrosine kinase binding” (GO:0030971, the 47th deregulated GO term in stage III) is the child of “protein tyrosine kinase binding” (GO:1990782, the 1st deregulated GO term in stage III). It can activate the PI3K-AKT pathway (the 1st, 2nd and 4th deregulated pathway in stage I, II, IV, respectively). The PI3K/AKT/mTOR pathway is frequently activated in EOCs [7] and leads to abnormal cell growth, proliferation and malignant transformation [8]. Deregulations in PI3K-AKT, protein tyrosine kinase binding, receptor tyrosine kinase binding and mTOR were among the most significantly deregulated functions detected in this study. Androgens can stimulate ovarian epithelial cells, resulting in increased proliferation and protection from apoptosis. Evidence has shown that androgen receptor is involved in the pathogenesis of ovarian cancer, and clinical trials using anti-androgens showed a response in relapsed ovarian cancer [9]. The role of AKAP13 in ovarian cancer is still unclear. However, evidence has shown it is a proto-oncogene that interacts with estrogen receptor alpha to regulate cell growth; it is also expressed in ovarian epithelial neoplasms [10]. Aberrant glycosylation and glycosphingolipid expression were associated oncogenic transformation [11]. Ganglioside levels can affect the motility of ovarian carcinoma cells [12] and regulate cell proliferation by affecting tyrosine kinase activity [13]. These commonly deregulated functions interacted with each other and the shared part of these most significantly deregulated functions was associated with cell cycle, cell proliferation or growth. Notably, the proportion of the cell cycle-related GO terms was prominently increased in stages III and IV. The “spindle pole”, “single-stranded DNA binding”, “spindle”, “damaging DNA binding”, and “structure-specific DNA binding” were the 7th, 9th, 11th, 12th and 15th most deregulated GO terms related to the cell cycle in stage III. The analysis of the deregulated canonical pathways also revealed consistent findings. 

One important feature of complex diseases such as SC is the aberrations in multiple gene functions and their interactions. However, the analysis of the *p* values for the most significant GO terms or canonical pathways did not provide information on the structure of SC pathogenesis. The co-recurrence of some significantly deregulated GO terms or canonical pathways implied the existence of interactions among these deregulated functions. To discover the members in the SC pathogenesis network, we performed a set analysis and EFA of these significant GO terms or pathways to identify the elements involved in the pathogenesis of SC, and the result showed that the most commonly deregulated functions between the GO terms and canonical pathways were related to the cell cycle. To detect the elements of the network involved in the carcinogenesis of SC among the 1454 GO terms, we executed the EFA and mapped the elements of the factors to the GO tree to further summarize them according to their parent-child GO hierarchy. The result showed that the cell cycle, programmed cell death, immune response, regulation of transcription and oxidoreductase activity were the most commonly deregulated functions involved in the pathogenesis of SC. The network reconstructed from the mutual information for these EFA elements showed extensive interactions among these deregulated functions. 

In addition to the EFA, the most important deregulated functions were investigated by tracing their rankings in the functionome from stage I to IV. These progressively deregulated GO terms, including cell cycle, immune response and oxidoreductase activity, showed gradually decreased function regularity and increased in ranking from stage I to IV; “mitotic cell cycle checkpoint” was the most import element among the cell cycle-related GO terms. 

Currently, the two-tier system classifying EOC to low-grade or high-grade cancer is widely accepted because it is reproducible [14]. In addition, based on the clinicopathological and molecular features, a dualistic model was proposed that divides EOCs into type I and II categories [15]. Type II EOC, which is mainly high-grade SC, exhibits impaired DNA damage repair and a more uncontrolled cell differentiation and aggressive behavior. TP53 was the primary molecular aberration observed in the pathogenesis of high-grade SC, which leads to deregulation of cell cycle control and increased mitotic figures, cell proliferation and aggressive behavior. However, most of the datasets in this study did not provide information regarding these classifications. Because high-grade SC constitutes 90% of the total SCs, it is reasonable to assume most of the samples in this study were high-grade SC or type II EOC. Our results from the functionome analysis were compatible with the behavior of type II, high-grade SC and the sequelae of TP53 aberration. In addition to the cell cycle, this model also detected numerous aberrant pathways reported in The Cancer Genome Atlas (TCGA) study [16], including the PI3K, NOTCH and forkhead box protein M1 (FOXM1) pathways, all of which were highly ranked on the list and showed statistical significance (Table S3). 

The workflow of analyzing microarray gene expression data usually consists of detecting the differentially expressed genes and then mapping them to the GO terms or pathways in an enrichment analysis to identify the aberrant functions. This approach will focus on the statistically significant genes or functions, but those genes that do not reach significance will be omitted. However, complex diseases, such as ovarian cancers, usually involve multiple genes or functional aberrations, as well as their interactions. To consider these features, we conducted this gene set-based study and investigated the pathogenesis of SC based on the “functionome”. The gene expression profiles were converted to orderings, and the functions were quantified by measuring the changes in the gene expression ordering changes among the genes in the gene sets defined by the GO terms or canonical pathways. Computing the changes in gene expression ordering in a gene set will consider the interactions of the gene elements in a gene set. In addition, functions are more easily understood than gene symbols, and converting tens of thousands of gene expression levels to approximately one thousand GSR indices will reduce the dimensions and noise of the data. This workflow is able to provide a more comprehensive and intuitive way to view the functionome and understand the pathogenesis of SC. The GSR model converts gene expression profiles to gene expression orderings in ordinal data; this data type will encounter less bias during the cross-platform integration of gene expression datasets than gene expression levels. This conversion makes it feasible for the GSR model to integrate the microarray gene expression datasets from different microarray platforms. 

This model had limitations. The first is that the GO terms and canonical pathway gene set databases did not define all human functions. For example, the GO term “cell cycle” (GO:0007049) has more than 8000 offspring. However, far fewer GO terms related to cell cycle were defined in the MSigDB, which might reduce the informativeness of this model. The second limitation is the detectability of this model. The GSR model converted gene expression levels to gene expression ordering. If the expression levels do not reach the detection levels, the GSR index will remain unchanged and aberrations will be missed. The third limitation is false positivity. Duplicated elements may exist in different gene sets and lead to false positive findings. For example, the 68th most significantly deregulated canonical pathway in the stage I group was “KEGG olfactory transduction”. The “olfactory transduction” function is apparently not involved in the carcinogenesis of SC. This false positivity resulted from the similar response of gene elements to G-protein transduction in the “KEGG olfactory transduction” gene set; however, G-proteins were shown to be involved in the carcinogenesis of SC using this model.

## 4. Materials and Methods

### 4.1. Workflow of Computing GSR Indices

The GSR index is computed by modified Differential Rank Conservation (DIRAC) [17], an algorithm measuring the ordering perturbations of gene elements in a gene set. In contrast to gene set perturbation, the GSR model quantifies the ordering changes of the gene elements in a gene set between two different phenotypes, such as cancer and the normal state in this study. The microarray gene expression profiles were downloaded from the GEO database in soft format, and then the gene expression levels were converted to the gene expression orderings using the gene sets defined by the GO terms or canonical pathway gene set databases. The GSR index was computed by measuring the differences in gene expression ordering in a gene set between the cancerous and the baseline gene set ordering template, which was defined as the most common gene expression ordering among the normal ovarian control samples. The baseline gene set ordering template for each gene set was established by pairwise comparison between the expression levels of two genes for all possible combinations of a gene pair. A gene set contains m genes *G* = {*G*_1_, …, *G_m_*} and the corresponding gene expression profile *E* = (*E*_1_, …, *E_m_*), *E_i_* denotes the expression level of gene *G_i_*. Each sample is labeled by a phenotype of a case (SC stage I–IV) or normal control group, respectively. The baseline gene set ordering template for each gene set is established by pairwise comparison between the expression levels of two genes for all possible combinations of a gene pair. The baseline gene ordering template **B** for a given gene set **G** is the binary vector composed of symbol “A” or “B”, where each component is “A” if the probabilities Pr(*E_i_* < *E_j_*|phenotype = control) > 0.5; or “B” if Pr(*E_i_* < *E_j_*|phenotype = control) ≤ 0.5. For the expression profile of a given sample ***e**_n_*, the GSR index R for a given gene set is the fraction of the *m* × (*m* − 1)/2 pairs for which the observed gene expression orderings within ***e**_n_* match the baseline gene ordering template **B**, namely, *R* = (number of “A”)/(*m* × (*m* − 1)/2). Establishment of the baseline gene set expression ordering templates and measurement of GSR indices were executed in the R environment; the code and the test datasets are available on the GitHub (https://github.com/carlzang/GSR-model.git).

### 4.2. Microarray Datasets, Gene Set Definition and Data Processing

The selection criteria for the downloaded microarray gene expression datasets were as follows: (1) both the case and normal control samples should originate from ovarian tissue; (2) the datasets should provide clear information about the diagnosis and stage of each sample; (3) a dataset was discarded if it resulted in less than 4000 common genes upon integration because this study utilized the common genes among the selected datasets; and (4) the gene expression profile was discarded if it contained missing data.

### 4.3. Statistical Analysis

The differences in the GSR indices between the four SC stages and the control groups were tested by the Mann-Whitney *U* test and corrected by multiple hypotheses using the false discovery rate (Benjamini-Hochberg procedure). The significance level was set at <0.05.

### 4.4. Classification and Prediction by Machine Learning

The GSR indices computed through the GO terms and canonical pathway gene sets were classified and predicted by SVM with kernlab [18], which is an R package for kernel-based machine-learning methods and was used to classify the patterns of the GSR indices with the following settings: kernel = “vanilladot” (linear kernel function) and type = “C-svc” (C classification). The performance of the classification and predictions by SVM were measured by five-fold cross-validation; the samples were randomly sampled and divided into five parts: four parts were used for training sets and the remaining part was used for the prediction. The performance of binary classification was assessed by sensitivity, specificity, accuracy and area under the curve (AUC). Sensitivity, specificity, accuracy and AUC were computed using the cumulative results of 10 repeated classifications. AUC was computed by an R package, pROC [19]. The performance of multiclass classification was assessed by the accuracy computed from the fraction of correct predictions within total prediction number. 

### 4.5. Hierarchical Clustering, Dendrogram and Heatmaps

All the GSR indices in each gene set and for each group were averaged then underwent hierarchical clustering with the function “heatmap.2” in R package “gplots” (version 2.17.0) as the default. This function executed the hierarchical clustering and drew the dendrogram and heatmaps. 

### 4.6. Set Analysis

All possible logical relations among the deregulated gene sets of the stage I–IV groups were evaluated by set analysis and displayed in a Venn diagram using the R package “VennDiagram” (version 1.6.16).

### 4.7. Exploratory Factor Analysis for the Deregulated GO Terms and Establishment of the GO Tree

The deregulated GO terms with a *p* value <0.05 were selected for the exploratory factor analysis (EFA). EFA was executed with the R package “psych” (version 1.5.8). The number of factors to be extracted was determined by the function “pa.parellel”. The factoring method used in this study was set to “pa” and the correlation matrix rotation method was “promax”. The tree of the deregulated GO terms was constructed and visualized in Portable Network Graphics (PNG) format constructed by the “RamiGO” [20], an R package providing functions that interact with the AmiGO 2 web server [21] and retrieves the GO trees.

### 4.8. Ranking Analysis

The importance of the given GO terms was evaluated by their rankings in the functionome at different stages during the progression of SC. To compare the rankings at different stages, we selected the GO terms with the following criteria: (1) *p* < 0.05; (2) The rank at stage IV was less than 200; and (3) the difference in the ranks between two consecutive stages was more than 15. The ranks of the selected GO terms were displayed on a line chart to show the paths of the changes in rankings from stage I to IV. 

### 4.9. Construction of the Interaction Network

The network was established with the mutual information based on entropy estimates from the k-nearest neighbor distances and Algorithm for the Reconstruction of Accurate Cellular Networks (ARACNE), and the interaction networks (multiplicative model) were reconstructed using the R package “parmigene” (version 1.0.2). The network was exported in the graph modeling language (GML) format and displayed on Cytoscape (version 3.3.0).

### 4.10. Detection of Differentially Expressed Genes in Ovarian Serous Carcinoma

To discover the DEGs in SC, we merged all microarray gene expression datasets and carried out integrative analysis. The gene expression levels were transformed and rescaled to cumulative proportion values from 0 (lowest expression) to 1 (highest expression) with an R package “YuGene” (version 1.1.5) for all samples in each dataset before integration. The DEGs were discovered using linear model computed with empirical Bayes analysis by the functions “lmFit” and “eBayes” provided by the R package “limna” (version 3.26.9).

## 5. Conclusions

By converting the gene expression levels into gene expression rankings through the gene ontology terms or canonical pathway gene set, the function defined by that gene set was quantified into a GSR index. In this study, we investigated the pathogenesis of SC using the functionome consisting of 1454 GO terms or 1330 canonical pathway-defined functions. We showed that the informativeness of the GSR indices was sufficient for accurate pattern recognition and classification, and the function regularity showed a stepwise deterioration, consistent with the severity of SC according to the four FIGO stages. Through a series of analyses using statistical methods, set analysis, EFA and ranking analysis, the results revealed that the core of SC pathogenesis was related to the cell cycle. The cell cycle began to be deregulated in stage I and worsened as the disease progressed. The pathogenesis of SC was complicated and involved aberrations in multiple functions and their interactions. In addition to the cell cycle, several other deregulated functions also participated in the network of SC pathogenesis, including channel activity, transport, binding, metabolism, cell differentiation, hormone response, protein kinase activity, oxidoreductase activity, GTPase activity, actin, cytoskeleton, chromosome, protein complex assembly, cell adhesion, catabolism, programmed cell death, cell proliferation, immune response, and regulation of transcription. 

## Figures and Tables

**Table 1:** Sample number and mean gene set regularity indices for each group. The table displayed the sample numbers, means and standard deviations (SDs) of the gene set regularity (GSR) indices for the four stages and the normal ovarian tissue controls computed through the gene ontology (GO) term gene sets. The gene expression profiles of the 136 normal ovarian tissue samples were utilized as the control group for the stage I–IV groups.

Stage	Case	Control	Total	Case Mean (SD)	Control Mean (SD)	*p* Value *
**I**	34	136	170	0.7425 (0.1511)	0.7752 (0.1370)	<0.05
**II**	39	136	175	0.7088 (0.1745)	0.7752 (0.1369)	<0.05
**III**	689	136	825	0.6483 (0.2007)	0.7738 (0.1548)	<0.05
**IV**	131	136	267	0.6197 (0.1922)	0.7737 (0.1413)	<0.05

SD: standard deviation; * Mann-Whitney *U* test.

**Table 2:** Accuracies of the binary and multiclass classifications and predictions by machine learning. This table displayed the performances of the binary (each stage group *vs.* control group) and multiclass classifications (the four stage groups + normal control group) and predictions by SVM using the GSR indices computed through the GO terms. The sensitivities, specificities, areas under the curves (AUCs), accuracies and standard deviation (SD) were measured by five-fold cross-validation. Each measurement was computed from 10 cumulative results of the repeated classifications and predictions.

Classification	Stage	Sensitivity (SD)	Specificity (SD)	Accuracy (SD)	AUC
Binary	**I**	0.9488 (0.0857)	1.0000 (0.0000)	0.9882 (0.0205)	0.9692
	**II**	0.9655 (0.0568)	1.0000 (0.0000)	0.9914 (0.0138)	0.9807
	**III**	0.9920 (0.0069)	0.9769 (0.0363)	0.9890 (0.0079)	0.9835
	**IV**	0.9929 (0.0149)	0.9961 (0.0121)	0.9943 (0.0091)	0.9942
Multiclass	**I**–**IV**	NA	NA	0.9038 (0.0054)	NA

AUC, area under the curve; SD, standard deviation; NA, not available.

**Table 3:** The 15 most deregulated gene ontology terms for the four stage groups ranked by their *p* values.

Ranking	Stage I	Stage II	Stage III	Stage IV
1	Calcium channel activity	lysosomal membrane	protein tyrosine kinase activity	lysosomal membrane
2	Cell maturation	vacuolar membrane	vitamin metabolic process	vacuolar membrane
3	Oxygen binding	regulation of actin filament length	oxidoreductase activity acting on the aldehyde or OXO group of donors	regulation of actin filament length
4	Secretin-like receptor activity	regulation of actin polymerization and/or depolymerization	regulation of actin filament length	regulation of cellular component size
5	Lysosomal membrane	regulation of cellular component size	regulation of actin polymerization and/or depolymerization	regulation of actin polymerization and/or depolymerization
6	Vacuolar membrane	amino acid derivative metabolic process	regulation of cellular component size	vacuolar part
7	Developmental maturation	response to hormone stimulus	spindle pole	cell division
8	Taste receptor activity	vacuolar part	homophilic cell adhesion	cytokinesis
9	Hematopoietin interferon class D200 domain Cytokine receptor binding	neuropeptide signaling pathway	single-stranded DNA binding	cell maturation
10	Cofactor transporter activity	G-protein coupled receptor binding	innate immune response	amino acid derivative metabolic process
11	Auxiliary transport protein activity	vitamin metabolic process	spindle	vitamin metabolic process
12	Hormone activity	steroid hormone receptor binding	damaged DNA binding	response to hormone stimulus
13	Organic anion transmembrane transporter activity	aromatic compound metabolic process	Rho protein signal transduction	calcium channel activity
14	Response to hormone stimulus	cell maturation	microtubule cytoskeleton	coenzyme binding
15	Potassium channel regulator activity	chaperone binding	structure specific DNA binding	developmental maturation

**Table 4:** The 15 most deregulated canonical pathways in the stage I–IV groups ranked by their *p* values.

Ranking	Stage I	Stage II	Stage III	Stage IV
1	Reactome CD28-dependent PI3K AKT signaling	Biocarta AKAP13 pathway	PID AR TF pathway	KEGG glycosphingolipid biosynthesis ganglio series
2	Biocarta AKAP13 pathway	Reactome CD28-dependent PI3K AKT signaling	KEGG glycosphingolipid biosynthesis ganglio series	Biocarta AKAP13 pathway
3	KEGG ascorbate and aldarate metabolism	Reactome PI3K events in ERBB4 signaling	Biocarta CK1 pathway	PID AR TF pathway
4	KEGG glycosphingolipid biosynthesis ganglio series	KEGG ascorbate and aldarate metabolism	Reactome COPI-mediated transport	Reactome CD28-dependent PI3K AKT signaling
5	Reactome signaling by NOTCH3	Reactome GPVI-mediated activation cascade	Reactome G0 and early G1	Reactome GPVI-mediated activation cascade
6	Reactome packaging of telomere ends	Biocarta MTA3 pathway	Reactome sphingolipid de novo biosynthesis	Reactome hormone-sensitive lipase HSL-mediated triacylglycerol hydrolysis
7	Reactome meiotic synapsis	Reactome GAB1 signalosome	KEGG cell cycle	Reactome termination of O glycan biosynthesis
8	KEGG retinol metabolism	Reactome PI3K AKT activation	Reactome DARPP 32 events	KEGG aldosterone-regulated sodium reabsorption
9	Reactome apoptotic cleavage of cell adhesion proteins	Reactome post-chaperonin tubulin folding pathway	Reactome meiotic synapsis	Reactome PI3K events in ERBB4 signaling
10	Reactome cytosolic sulfonation of small molecules	KEGG glyoxylate and dicarboxylate metabolism	PID AR pathway	KEGG inositol phosphate metabolism
11	Reactome digestion of dietary carbohydrate	Reactome GABA synthesis release reuptake and degradation	Reactome neurotransmitter release cycle	Reactome G0 and early G1
12	Reactome peptide ligand binding receptors	Reactome packaging of telomere ends	PID AJDISS 2 pathway	KEGG acute myeloid leukemia
13	Reactome synthesis of PIPS at the plasma membrane	KEGG glycosphingolipid biosynthesis ganglio series	Reactome signaling by Rho GTPases	KEGG tryptophan metabolism
14	Reactome xenobiotics	PID TGFBR pathway	KEGG progesterone-mediated oocyte maturation	Reactome downregulation of ERBB2 ERBB3 signaling
15	SA TRKA receptor	Reactome adenylate cyclase inhibitory pathway	Reactome trans Golgi network vesicle budding	Reactome meiotic synapsis

**Table 5:** Top 10 down-regulated differentially expressed genes for serous carcinoma.

Gene Symbol	Alias	Related GO Terms or Pathways	*p* Value
AOX1	Aldehyde Oxidase 1	Catalytic activity (GO:0003824)	3.51 × 10^−133^
		Aldehyde oxidase activity (GO:0004031)	
		Small molecule metabolic process (G0:0044281)	
EIF3F	Eukaryotic Translation Initiation Factor 3, Subunit F	Translation initiation factor activity (GO:0003743)	2.00 × 10^−132^
		Protein binding (GO:0005515)	
		TRanslation (GO:0006412)	
		Eukaryotic translation initiation (Reactome)	
		Activation of the mRNA upon binding of the cap-binding complex and eIFs and subsequent binding to 43S (Reactome)	
DFNA5	Deafness, Autosomal Dominant 5	Apoptotic process (GO:0006915)	1.26 × 10^−128^
		Negative regulation of cell proliferation (GO:0008285)	
		Positive regulation of intrinsic apoptotic signaling Pathway (GO:2001244)	
PTGIS	Prostaglandin I2 (Prostacyclin) Synthase	Monooxygenase activity (GO:0004497)	6.85 × 10^−125^
		Protein binding (GO:0005515)	
		Oxidoreductase activity acting on paired donors with Incorporation or reduction of molecular oxygen (GO:0016705)	
TSPAN5	Tetraspanin 5	Positive regulation of Notch signaling pathway (GO:0045747)	7.08 × 10^−124^
		Protein maturation (GO:0051604)	
BAMBI	BMP and Activin Membrane-Bound Inhibitor	Positive regulation of cell proliferation (GO:0008284)	2.13 × 10^−108^
		Transforming growth factor β receptor signaling pathway (GO:0007179)	
		TGF-β receptor signaling (PID)	
SPOCK1	Sparc/Osteonectin, Cwcv and Kazal-Like Domains Proteoglycan (Testican) 1	Serine-type endopeptidase inhibitor activity (GO:0004867)	2.13 × 10^−108^
		Cysteine-type endopeptidase inhibitor activity (GO:0004869)	
		Calcium ion binding (GO:0005509)	
		Protein binding (GO:0005515)	
		Metalloendopeptidase inhibitor activity (GO:0008191)	
GFPT2	Glutamine-Fructose-6-Phosphate Transaminase 2	Glutamine-fructose-6-phosphate transaminase (isomerizing) activity (GO:0004360)	8.91 × 10^−107^
		Carbohydrate binding (GO:0030246)	
		Amino sugar and nucleotide sugar metabolism (KEGG)	
C21orf62	Chromosome 21 Open Reading Frame 62	Unclear	1.35 × 10^−106^
FLRT2	Fibronectin Leucine Rich Transmembrane Protein 2	Receptor signaling protein activity (GO:0005057)	5.29 × 10^−104^
		Protein binding (GO:0005515)	
		Fibroblast growth factor receptor signaling pathway (GO:0008543)	
		Cell adhesion (GO:0007155)	

**Table 6:** Top 10 up-regulated differentially expressed genes for serous carcinoma.

Gene Symbol	Alias	Related GO Terms or Pathways	*p* Value
C14orf2	Chromosome 14 Open Reading Frame 2	unclear	8.15 × 10^−78^
COX6B1	Cytochrome C Oxidase Subunit VIb Polypeptide 1	transcriptional regulation by TP53(Reactome)	2.59 × 10^−66^
		gene expression (Reactome)	
		transcription initiation from RNA polymerase II	
		promoter ( GO:0006367)	
		gene expression (GO:0010467)	
TRIAP1	TP53 Regulated Inhibitor of Apoptosis 1	p53 binding (GO:0002039)	3.44 × 10^−65^
		DNA damage response signal transduction by p53 class mediator resulting in cell cycle arrest (GO:0006977)	
		DNA damage response signal transduction by p53 class mediator (GO:0030330)	
		negative regulation of apoptotic process (GO:0043066)	
RBX1	Ring-Box 1, E3 Ubiquitin Protein Ligase	contributes to ubiquitin-protein transferase activity (GO:0004842)	9.37 × 10^−63^
		DNA repair (GO:0006281)	
		MAPK cascade (GO:0000165)	
		signaling by ERBB2 (Reactome)	
		RAF/MAP kinase cascade (Reactome)	
CGRRF1	Cell Growth Regulator with Ring Finger Domain 1	response to stress (GO:0006950)	1.25 × 10^−61^
		cell cycle arrest (GO:0007050)	
		negative regulation of cell proliferation (GO:0008285)	
LSM6	LSM6 Homolog, U6 Small Nuclear RNA and MRNA Degradation Associated	cytoplasmic mRNA processing body (GO:0000932)	6.16 × 10^−60^
		spliceosomal complex (GO:0005681)	
		U6 snRNP (GO:0005688)	
		nucleolus (GO:0005730)	
		small nucleolar ribonucleoprotein complex (GO:0005732)	
		deadenylation-dependent mRNA decay (Reactome)	
COX5A	Cytochrome C Oxidase Subunit Va	cytochrome-c oxidase activity (GO:0004129)	1.71 × 10^−59^
		transcriptional regulation by TP53 (Reactome)	
		mitochondrial electron transport; cytochrome c to oxygen (GO:0006123)	
		transcription initiation from RNA polymerase II promoter (GO:0006367)	
		gene expression (GO:0010467)	
TIMM8B	Translocase of Inner Mitochondrial Membrane 8 Homolog B (Yeast)	protein targeting to mitochondrion (GO:0006626)	1.54 × 10^−58^
		protein transport (GO:0015031)	
		cellular protein metabolic process (GO:0044267)	
		chaperone-mediated protein transport (GO:0072321)	
SNX6	Sorting Nexin 6	type I transforming growth factor beta receptor binding (GO:0034713)	1.62 × 10^−58^
		phosphatidylinositol binding (GO:0035091)	
		protein homodimerization activity (GO:0042803)	
		TGF-β receptor signaling pathway (Reactome)	
		negative regulation of epidermal growth factor-activated receptor activity (GO:0007175)	
		negative regulation of transforming growth factor β receptor signaling pathway (GO:0030512)	
IER3IP1	Immediate Early Response 3 Interacting Protein 1	regulation of fibroblast apoptotic process (GO:2000269)	1.88 × 10^−58^
		endoplasmic reticulum (GO:0005783)	

**Table 7:** Top 20 progressively deregulated genes from stage I to IV.

Gene Symbol	Alias	Related GO Terms or Pathways
UFC1	Ubiquitin-Fold Modifier Conjugating Enzyme 1	protein binding (GO:0005515)
		response to endoplasmic reticulum stress (GO:0034976) protein ufmylation (GO:0071569)
SOX12	SRY (Sex Determining Region Y)-Box 12	transcription regulatory region sequence-specific DNA binding (GO:0000976)
		transcriptional activator activity, RNA polymerase II core promoter proximal region sequence-specific binding (GO:0001077)
		RNA polymerase II transcription coactivator activity (GO:0001105)
		DNA binding (GO:0003677)
		molecular mechanisms of cancer (QIAGEN)
APOC3	Apolipoprotein C-III	phospholipid binding (GO:0005543)
		cholesterol binding (GO:0015485)
		enzyme regulator activity (GO:0030234)
		lipase inhibitor activity (GO:0055102)
		signal transduction (Reactome)
		G-protein coupled receptor signaling pathway (GO:0007186)
RAB11FIP2	RAB11 Family Interacting Protein 2 (Class I)	Rab GTPase binding (GO:0017137)
		protein kinase binding (GO:0019901)
		protein homodimerization activity (GO:0042803)
PCOLCE2	Procollagen C-Endopeptidase Enhancer 2	protein binding (GO:0005515)
		collagen binding (GO:0005518)
		heparin binding (GO:0008201)
		peptidase activator activity (GO:0016504)
		collagen formation (Reactome)
		positive regulation of peptidase activity (GO:0010952)
STAT2	Signal Transducer and Activator Of Transcription 2, 113 kDa	DNA binding (GO:0003677)
		transcription factor activity, sequence-specific DNA binding (GO:0003700)
		signal transducer activity (GO:0004871)
		Jak-STAT signaling pathway (KEGG)
		transcription, DNA-templated (GO:0006351)
		regulation of transcription, DNA-templated (GO:0006355)
		regulation of transcription from RNA polymerase II promoter (GO:0006357)
AR	Androgen Receptor	RNA polymerase II core promoter proximal region sequence-specific DNA binding (GO:0000978)
		RNA polymerase II transcription factor binding (GO:0001085)
		DNA binding (GO:0003677)
		chromatin binding (GO:0003682)
		signaling by Rho GTPases (Reactome)
		regulation of transcription, DNA-templated (GO:0006355)
INSIG2	Insulin Induced Gene 2	transcription factor binding (GO:0008134)
		regulation of cholesterol biosynthesis by SREBP (Reactome)
		cholesterol biosynthetic process (GO:0006695)
		response to sterol depletion (GO:0006991)
		cholesterol metabolic process (GO:0008203)
		negative regulation of steroid biosynthetic process (GO:0010894)
POLR2G	Polymerase (RNA) II (DNA Directed) Polypeptide G	nucleic acid binding (GO:0003676)
		single-stranded DNA binding (GO:0003697)
		single-stranded RNA binding (GO:0003727)
		translation initiation factor binding (GO:0031369)
		mRNA splicing, via spliceosome (GO:0000398)
		DNA repair (GO:0006281)
CHODL	Chondrolectin	carbohydrate binding (GO:0030246)
		regulation of neuron projection development (GO:0010975)
		perinuclear region of cytoplasm (GO:0048471)
COL4A1	Collagen, Type IV, Alpha 1	extracellular matrix structural constituent (GO:0005201)
		protein binding (GO:0005515)
		extracellular matrix constituent conferring elasticity (GO:0030023)
		platelet-derived growth factor binding (GO:0048407)
		focal adhesion (KEGG)
		patterning of blood vessels (GO:0001569)
		receptor-mediated endocytosis (GO:0006898)
RAB9A	RAB9A, Member RAS Oncogene Family	GTPase activity (GO:0003924)
		GTP binding (GO:0005525)
		GDP binding (GO:0019003)
		signal transduction (GO:0007165)
		small GTPase-mediated signal transduction (GO:0007264)
EN1	Engrailed Homeobox 1	RNA polymerase II core promoter proximal region sequence-specific DNA binding (GO:0000978)
		transcriptional repressor activity, RNA polymerase II core promoter proximal region sequence-specific binding (GO:0001078)
		DNA binding (GO:0003677)
		sequence-specific DNA binding (GO:0043565)
ATP1B1	ATPase, Na+/K+ Transporting, Beta 1 Polypeptide	ATPase activator activity (GO:0001671)
		response to hypoxia (GO:0001666)
		potassium ion transport (GO:0006813)
		sodium ion transport (GO:0006814)
		cellular calcium ion homeostasis (GO:0006874)
GNAT1	Guanine Nucleotide Binding Protein (G Protein), Alpha Transducing Activity Polypeptide 1	acyl binding (GO:0000035)
		G-protein coupled receptor binding (GO:0001664)
		GTPase activity (GO:0003924)
		signal transducer activity (GO:0004871)
		activation of the phototransduction cascade (Reactome)
		G-protein coupled receptor signaling pathway (GO:0007186)
PDCD6IP	Programmed Cell Death 6 Interacting Protein	SH3 domain binding (GO:0017124)
		proteinase activated receptor binding (GO:0031871)
		protein homodimerization activity (GO:0042803)
		protein dimerization activity (GO:0046983)
		cell separation after cytokinesis (GO:0000920)
		apoptotic process (GO:0006915)
		regulation of centrosome duplication (GO:0010824)
PDHB	Pyruvate Dehydrogenase (Lipoamide) Beta	catalytic activity (GO:0003824)
		pyruvate dehydrogenase activity (GO:0004738)
		glucose metabolic process (GO:0006006)
		acetyl-CoA biosynthetic process from pyruvate (GO:0006086)
		pyruvate metabolic process (GO:0006090)
		tricarboxylic acid cycle (GO:0006099)
GCNT3	Glucosaminyl (N-Acetyl) Transferase 3, Mucin Type	acetylglucosaminyltransferase activity (GO:0005975) carbohydrate metabolic process (GO:0008375)
		protein O-linked glycosylation (GO:0006493)
		post-translational protein modification (GO:0043687)
FXYD3	FXYD Domain Containing Ion Transport Regulator 3	ion channel activity (GO:0005216)
		chloride channel activity (GO:0005254)
		sodium channel regulator activity (GO:0017080)
		ATPase binding (GO:0051117)
CHGA	Chromogranin A	protein binding (GO:0005515)
		Peptide hormone biosynthesis (Reactome)
		Androgen biosynthesis (Reactome)
		Signaling by GPCR (Reactome)

## Supplementary Materials

Supplementary materials can be found at http://www.mdpi.com/1422-0067/17/6/886/s1.

## Abbreviations

SC	Serous carcinoma
FIGO	Federation of Gynecologists and Obstetrics
GO	Gene ontology
GSR	Gene set regularity
EFA	Exploratory factor analysis
EOC	Epithelial ovarian cancers
MSigDB	Molecular Signatures Database
KEGG	Kyoto Encyclopedia of Genes and Genomes
PID	Pathway Interaction Database
GEO	Gene Expression Omnibus
SD	Standard deviation
SVM	Support vector machine
AUC	Area under curve
PI3K-AKT	Phosphoinositide 3-kinase-AKT
AKAP13	A Kinase Anchor Protein 13
AR TR	Androgen receptor transcription factor
EIF2	Eukaryotic initiation factor-2
mTOR	Mammalian target of rapamycin
FOXM1	Forkhead box protein M1
DIRAC	Differential Rank Conservation
PNG	Portable Network Graphics
ARACNE	Algorithm for the Reconstruction of Accurate Cellular Networks
GML	graph modeling language
